# Pulmonary exacerbations in primary ciliary dyskinesia

**DOI:** 10.1007/s00431-026-07363-x

**Published:** 2026-09-07

**Authors:** Dvir Gatt, Inbal Golan-Tripto, Aviv Goldbart, Guy Hazan

**Affiliations:** 1 The Faculty of Health Sciences, Ben-Gurion University of the Negev, Beer Sheva, Israel; 2 Pediatric Pulmonology Unit, Saban Children’s Hospital, Soroka University Medical Center, Beer Sheva, Israel

**Keywords:** Primary ciliary dyskinesia, Pulmonary exacerbations, Bronchiectasis, Lung function, Airway clearance, Azithromycin

## Abstract

Primary ciliary dyskinesia (PCD) is a rare inherited disorder of motile ciliary dysfunction characterized by impaired mucociliary clearance, chronic sino-pulmonary disease, and progressive bronchiectasis. Pulmonary exacerbations (PEx) are a major contributor to morbidity, lung function decline, and healthcare utilization in PCD, yet historically have been poorly defined and understudied. This review summarizes current evidence regarding the definition, epidemiology, pathophysiology, clinical impact, and management of PEx in PCD, with emphasis on recent advances that are beginning to establish a disease-specific evidence base. Recent international consensus efforts have proposed standardized definitions for PEx, although significant heterogeneity remains across clinical studies. Prospective cohort data demonstrate that PEx occur frequently across all age groups, including infancy, and are associated with impaired lung function recovery, structural lung damage, and increased disease severity, particularly in patients with chronic *Pseudomonas aeruginosa* infection. Emerging biomarkers such as lung clearance index and exhaled breath analysis using volatile organic compounds may improve prediction and monitoring of exacerbations. Management strategies currently rely largely on extrapolation from cystic fibrosis and non-cystic fibrosis bronchiectasis and include prompt antibiotic therapy, intensified airway clearance, and supportive care. However, recent randomized controlled trials, including BESTCILIA and CLEAN-PCD, have provided the first high-quality evidence supporting maintenance azithromycin therapy and airway hydration strategies in PCD. Despite these advances, important gaps remain regarding optimal exacerbation definitions, treatment duration, predictors of incomplete recovery, and the role of anti-inflammatory and targeted therapies.

*Conclusion*: Continued international collaboration, validated outcome measures, and development of mechanism-based therapies will be essential to improve long-term outcomes for patients with PCD.

**Table:** 

**What is Known:** • *Primary ciliary dyskinesia (PCD) is a rare inherited disorder of motile ciliary dysfunction causing chronic sino-pulmonary disease, recurrent respiratory infections, and progressive bronchiectasis from early childhood.* • *Pulmonary exacerbations (PEx) are a major driver of morbidity and lung function decline in PCD, but have historically been inconsistently defined and understudied, with management largely extrapolated from cystic fibrosis and non-CF bronchiectasis. Recent international consensus efforts have proposed standardized definitions for PEx, although significant heterogeneity remains across clinical studies.*	
**What is New:** • *Prospective international cohort data (Schreck et al. 2026) establish a PEx incidence of approximately 3.1 per person per year across all age groups, with higher risk in adult females and Pseudomonas aeruginosa–colonized patients. PEx are frequent even in the first 2 years of life. Pulmonary exacerbations cause substantial acute loss in forced expiratory volume in 1 s (FEV1), and approximately 20–25% of events do not recover to baseline lung function.* • *The BESTCILIA trial provides the first high-quality evidence that maintenance azithromycin reduces PEx frequency by 55% in PCD, and the CLEAN-PCD trial provides proof-of-concept for airway rehydration (ENaC blockade + hypertonic saline) as a PCD-specific therapeutic strategy.Novel anti-inflammatory therapies (DPP1 inhibitors, neutrophil elastase inhibitors), inhaled biologics, and gene-directed strategies are entering the PCD therapeutic pipeline and may transform future PEx prevention.*

**What is Known:**

• *Primary ciliary dyskinesia (PCD) is a rare inherited disorder of motile ciliary dysfunction causing chronic sino-pulmonary disease, recurrent respiratory infections, and progressive bronchiectasis from early childhood.*

• *Pulmonary exacerbations (PEx) are a major driver of morbidity and lung function decline in PCD, but have historically been inconsistently defined and understudied, with management largely extrapolated from cystic fibrosis and non-CF bronchiectasis. Recent international consensus efforts have proposed standardized definitions for PEx, although significant heterogeneity remains across clinical studies.*

**What is New:**

• *Prospective international cohort data (Schreck et al. 2026) establish a PEx incidence of approximately 3.1 per person per year across all age groups, with higher risk in adult females and Pseudomonas aeruginosa–colonized patients. PEx are frequent even in the first 2 years of life. Pulmonary exacerbations cause substantial acute loss in forced expiratory volume in 1 s (FEV1), and approximately 20–25% of events do not recover to baseline lung function.*

• *The BESTCILIA trial provides the first high-quality evidence that maintenance azithromycin reduces PEx frequency by 55% in PCD, and the CLEAN-PCD trial provides proof-of-concept for airway rehydration (ENaC blockade + hypertonic saline) as a PCD-specific therapeutic strategy.Novel anti-inflammatory therapies (DPP1 inhibitors, neutrophil elastase inhibitors), inhaled biologics, and gene-directed strategies are entering the PCD therapeutic pipeline and may transform future PEx prevention.*

## Introduction

Primary ciliary dyskinesia (PCD) is a rare autosomal recessive disorder caused by pathogenic variants in approximately 60 genes encoding proteins essential for ciliary structure and function [1–3]. The estimated prevalence is approximately 1 in 7500 individuals [4, 5], although the true burden is likely significantly higher owing to longstanding diagnostic delay [6]. The fundamental defect, dysfunction of motile cilia lining the respiratory tract, middle ear, paranasal sinuses, and reproductive organs, results in chronic sino-oto-pulmonary disease characterized by daily wet cough, recurrent respiratory tract infections, otitis media with effusion, and bronchiectasis that develops progressively from early childhood [1, 7].

Pulmonary exacerbations (PEx) are a major driver of morbidity and healthcare use in PCD. They cause acute lung function decline and contribute to structural lung damage. PEx require prolonged courses of antibiotics and drive healthcare utilization [8–10]. Yet despite their importance, historically, PEx in PCD were inconsistently defined and understudied, with management extrapolated from cystic fibrosis and non-CF bronchiectasis guidelines [11]. Research was constrained by the rarity of the disease, phenotypic heterogeneity, lack of validated outcome measures, and the consequent paucity of clinical trials [7, 11].

This landscape is now changing. Consensus definitions exist, multicenter randomized controlled trials have been completed, and important observational cohort data have characterized exacerbation frequency, lung function impact, and microbiology in PCD populations across all age groups. The purpose of this review is to synthesize this evidence for the general pediatrician and to identify the clinical and research priorities that remain unmet.

## Defining pulmonary exacerbations

A major barrier to progress in PCD PEx research has been the absence of a universally accepted definition. This has also been a longstanding concern in CF, where several definitions for PEx exist without a single accepted standard [12–15], largely due to variability in presenting symptoms and the reliance of many definitions on an “action-based” approach, meaning a physician intervention such as initiation of systemic antibiotic [16]. The initial common approach was to define PEx as increase in respiratory symptoms, treated with systemic antibiotics [17, 18] (Table 1).

**Table 1:** Definitions of pulmonary exacerbations in primary ciliary dyskinesia: historical and current frameworks

Source/year	Framework/context	Criteria/items required	Time element	Setting	Key notes
***Consensus/guidance documents (not age-specific)***					
Shapiro et al. 2016—PCD Foundation Consensus (Pediatr Pulmonol)	PCD foundation clinical consensus	Respiratory exacerbation: new or worsening respiratory symptoms; treatment decisions based on clinical judgment; 2–3-week antibiotic courses recommended	None	Clinical practice	Most influential North American clinical guidance document. Not a specific numerical definition; management-focused rather than research-focused
Lucas et al. 2019—International consensus (ERJ Open Res)	Delphi expert consensus (PCD-specific)	≥ 3 of 7 criteria: (1) Increased cough (2) Change in sputum volume/color (3) Increased SOB (4) Decision to start or change antibiotics (5) Malaise, tiredness, fatigue, lethargy (6) New or increased hemoptysis (7) Fever > 38°	None (acknowledged limitation)	Research (clinical trials in PCD)	First and currently most widely used PCD-specific consensus definition. Developed by 30 multidisciplinary experts + patients via modified Delphi process. Awaits prospective validation
Kos et al. 2024—BEAT-PCD Core Outcomes (ERJ Open Res)	International consensus on outcomes	PEx identified as one of four core outcomes for PCD intervention studies; no single definition threshold agreed upon	N/A	Research (clinical trials in PCD)	Endorses PEx as essential outcome measure. Notes ongoing lack of consensus on precise definition threshold
***Pediatric-only studies***					
Ratjen et al. 2016 (Eur Respir J)	PCD pediatric observational cohort (age 5.8–17.9 years)	Increase in respiratory symptoms treated with systemic antibiotics	None	Research-clinical (Canada, Hospital for Sick Children)	The first study to compare airway inflammation in CF and PCD patients in response to treatment of pulmonary exacerbations
Sunther et al. 2016 (Pediatr Pulmonol)	PCD pediatric observational cohort (age 6–16.2 years)	Increased respiratory symptoms requiring systemic antibiotic (IV)	None	Clinical (UK, pediatric)	IV treatment–based definition; first paper reporting FEV_1_ recovery trajectories in PCD children
Gatt et al. 2023 (Pediatr Pulmonol)—iPEx study	PCD pediatric observational cohort (median age 14.8 years)	Increased respiratory symptoms treated with IV antibiotics (iPEx)	IV treatment episode	Clinical (Canada, Hospital for Sick Children)	IV treatment–defined; first systematic characterization of FEV_1_ trajectories during IV PEx in PCD
Gatt et al. 2025 (Ann Am Thorac Soc)—oPEx study	PCD pediatric observational cohort (mean age at oPEx 12.5 years)	Increase in baseline symptoms with physician decision to treat with systemic antibiotics (oral or IV); oPEx = oral antibiotic treatment	Antibiotic course	Clinical (Canada, Hospital for Sick Children)	Action-based; includes spirometry criterion: FEV_1_ ≤ 90% of stable baseline used to define lung function non-recovery. Largest oPEx dataset in PCD (297 events)
McCoy J, et al. 2026 (Pediatr Pulmonol) [19]	PCD pediatric observational cohort	Increase in baseline symptoms with physician decision to treat with systemic antibiotics	None	Clinical (Canada, Hospital for Sick Children)	Studied impact of nutritional status on PEx outcomes
Kumar M et al. 2026 (Pediatr Pulmonol)	PCD pediatric observational cohort (ages 0–2 years)	Increase in baseline symptoms with physician decision to treat with systemic antibiotics	None	Clinical (two PCD centers, Canada)	PEx are also common during the first 2 years of life despite very early diagnosis
***Adult-only studies***					
Shah et al. 2016 (Eur Respir J)	PCD observational cohort (151 adult patients)	Action-based: systemic antibiotic initiation (oral or IV) for respiratory symptoms	None	Clinical (real-world, UK PCD center)	Pre-consensus definition; illustrates action-based approach in PCD prior to Lucas 2019
Paff et al. 2017—RCT (Eur Respir J)	PCD RCT (22 adult patients)	Increase in respiratory symptoms requiring antibiotic treatment (oral or IV)	None specified	Research (PCD-specific RCT)	Used in the first PCD-specific HTS RCT; action-based definition. Exacerbation rate a secondary endpoint
***Mixed pediatric* + *adult studies***					
Cohen-Cymberknoh et al. 2017 (Respir Med)	PCD observational cohort (115 pediatric patients and 102 adults)	Respiratory exacerbation requiring antibiotic treatment (IV or oral); ≥ 2/year defined “frequent”	None	Clinical (Israel PCD registry)	Action-based; ≥ 2 annual exacerbations used as severity threshold; linked to Pseudomonas colonization outcomes
Kobbernagel et al. 2020—BESTCILIA Trial (Lancet Respir Med)	PCD-specific RCT (90 patients, 64 under 22 years)	Exacerbation defined as any respiratory tract symptoms leading to initiation of systemic antibiotics OR ≥ 10% FEV_1_ decline	Duration of antibiotic course	Research (multicenter RCT)	Action-based operational definition (antibiotic initiation). Primary outcome was exacerbation rate
Piatti et al. 2020 (BMC Pediatr)	PCD retrospective cohort (37 adult patients and 21 children)	Exacerbation requiring antibiotic treatment; ≥ 2/year = frequent exacerbator subgroup	None	Clinical (Italy, mixed pediatric/adult)	Used same frequency threshold as Cohen-Cymberknoh 2017; associated with CT severity scores
Chang et al. 2022 —REPEAT Trial protocol (BMJ Open Respir Res)	PCD-specific RCT	≥ 2 exacerbations in preceding 18 months (eligibility); exacerbation defined as increase in respiratory symptoms treated with antibiotics	Antibiotic course	Research (multicenter RCT)	Combines frequency threshold for eligibility with action-based event definition; mirrors BESTCILIA approach
Schreck et al. 2026—Living with PCD Cohort (ERJ Open Res)	PCD prospective cohort: 660 patients (408 adults, 57 adolescents, 195 children)	Self-reported: increased respiratory symptoms in past 7 days (online questionnaire); proxy for PEx	7-day symptom window	Research/epidemiologic (international)	Patient-reported, time-anchored definition enabling prospective incidence data. 1,026 PEx in 660 participants over 2 years
Anagnostopoulou et al. 2026 (Front Mol Biosci)	PCD prospective study (271 patients, all ages): genetically confirmed diagnosis	Three definitions: (1) Lucas et al. international consensus (2) If the patient started or changed antibiotic treatment (3) Self-reported	Patient reported on PEx symptoms during “the last month”	Research/epidemiologic (international)	Differences in PEx frequency according to definition used. Female sex and autumn season were associated with higher number of PEx, independent of the definition used

*CF* cystic fibrosis, *ENaC* epithelial sodium channel, *FEV*_*1*_ forced expiratory volume in 1 s, *HTS* hypertonic saline, *iPEx* intravenously treated pulmonary exacerbation, *IV* intravenous, *LCI* lung clearance index, *oPEx* orally treated pulmonary exacerbation, *PCD* primary ciliary dyskinesia, *PEx* pulmonary exacerbation, *QOL* quality of life, *SOB* shortness of breath

Symptom duration is not specified as a criterion in most definitions listed above. This reflects the fact that symptom duration varies considerably according to disease severity, genotype, chronic infections, and treatment adherence, making a universal minimum-duration threshold difficult to establish across the heterogeneous PCD population

### The Lucas et al. (2019) international consensus

The most widely adopted research definition was developed by Lucas et al. through a modified Delphi process involving 30 multidisciplinary experts and patient representatives [20]. It requires the presence of three or more of the following seven criteria: (1) increased cough, reflecting heightened airway irritation and secretion burden; (2) a change in sputum volume and/or color; (3) increased dyspnea as perceived by the patient or parent, capturing subjective respiratory distress; (4) a clinician’s decision to start or change antibiotic treatment for respiratory symptoms, an action-based criterion reflecting real-world clinical judgment; (5) malaise, tiredness, fatigue, or lethargy; (6) new or increased hemoptysis; and (7) temperature above 38 °C, indicating a systemic inflammatory or infective response. Notably, no time element is specified, a limitation acknowledged by the authors, who proposed that the definition be validated in prospective studies.

Other frameworks (PCD Foundation, European Respiratory Society bronchiectasis statement) vary in criteria and severity thresholds [11, 21]. This definitional heterogeneity carries direct practical consequences. Because studies and treatment centers apply different combinations of clinical criteria, reported PEx incidence and outcome data are not always directly comparable across cohorts, complicating pooled analyses and evidence synthesis. In clinical trials, the choice of PEx as an endpoint is similarly constrained: without an agreed, validated threshold, investigators face uncertainty in selecting outcome definitions and calculating sample sizes.

A further limitation, highlighted by Schreck et al., is that clinically relevant exacerbations may not always be captured by these criteria [10]. Patients with established PCD frequently self-manage mild exacerbations conservatively or by using previously prescribed antibiotics without contacting their care team; observational incidence estimates may therefore underrepresent the true clinical burden of PEx. In addition, patients with more advanced disease and persistent baseline respiratory symptoms often have difficulty distinguishing acute symptom worsening from their chronic baseline status, further complicating both case ascertainment and the assessment of exacerbation duration. A related practical limitation is that one of the most important criteria, a change in sputum volume and/or color, is difficult to apply in young children, many of whom are unable to expectorate, adding a further source of variability to definitional heterogeneity across the pediatric age range. Collectively, these variations highlight the need for a validated, standardized, and clinically applicable definition, ideally incorporating patient-reported outcomes*.*

### Clinical trial definitions

Clinical trials (BESTCILIA, REPEAT) have used heterogeneous operational definitions based on antibiotic initiation, symptom change, or lung function decline [22, 23]. Furthermore, after the consensus definition on PEx, the BEAT-PCD published another consensus statement that identified PEx as one of four core outcomes for intervention studies in PCD [9] (Table 2). However, consensus on a precise definition threshold was not achieved. These differences emphasize the practical challenges of defining exacerbations in a heterogeneous disease, in which the clinical phenotype varies by age, genotype, and baseline symptom burden, such that no single symptom-based threshold is likely to perform equally well across the full spectrum of patients, underscoring the need for a standardized definition [9, 11].

**Table 2:** Key studies in primary ciliary dyskinesia with data relevant to pulmonary exacerbations. Studies listed in chronological order. Included if they used pulmonary exacerbation as a primary or secondary outcome, reported exacerbation rates/predictors, characterized lung function during/after PEx, or were randomized trials with PEx as an endpoint in PCD

Author/year/journal	Study design	*N*	Age range	PEx definition used	Key PEx-related findings	Key limitations
Sunther et al. 2016 Pediatr Pulmonol	Retrospective cohort	30 iPEx events	Children (median 9.5 yrs)	IV antibiotics for increased respiratory symptoms	77% (23/30) recovered to baseline FEV_1_ within 3 months. First published data on FEV_1_ recovery trajectories in PCD children after iPEx	Small sample; single UK center; retrospective; only IV exacerbations included
Shah et al. 2016 Eur Respir J	Longitudinal observational cohort	*n* = 119 (adults)	Adults (mean 34 yrs)	Antibiotic courses for respiratory symptoms	Mean 1.2 exacerbation courses/year. Pseudomonas aeruginosa colonization independently associated with greater exacerbation frequency and worse FEV_1_	Retrospective; adult-only; single UK PCD center; inconsistent definition
Cohen-Cymberknoh et al. 2017 Respir Med	Retrospective cohort	*n* = 78	Mixed (children + adults; mean 19.8 yrs)	IV or oral antibiotic treatment for respiratory symptoms; ≥ 2/yrs = frequent	Pseudomonas colonization in 51.4% of frequent exacerbators vs. 19.4% in infrequent. Chronic Pseudomonas independently associated with more hospitalizations, lower FEV_1_, more bronchiectasis	Retrospective; single Israeli center; inconsistent PEx definition; mixed age group
Paff et al. 2017 (HTS RCT) Eur Respir J	Randomized controlled trial (crossover)	*n* = 24	≥ 18 yrs (adults)	Antibiotic initiation for respiratory symptoms (secondary outcome)	No significant difference in primary QOL outcome (SGRQ total score). One secondary subscale (QoL-B Health Perception) improved with HTS. No significant difference in exacerbation rate. First PCD-specific RCT with exacerbation as outcome	Small sample; short duration (12 weeks); crossover design; adult-only; underpowered for exacerbation outcomes
Piatti et al. 2020 BMC Pediatr	Retrospective cohort	*n* = 58 (37 adults, 21 children)	Mixed ages (mean 25 yrs)	Antibiotic courses; ≥ 2/year = frequent exacerbator	Frequent exacerbators had significantly lower FEV_1_ (55% vs. 76%), more bronchiectasis lobes, worse CT Bhalla scores, and higher prevalence of chronic Pseudomonas (33.3% vs 13.6%). Exacerbation frequency and Pseudomonas colonization independently associated with structural/functional severity	Retrospective; small sample; single Italian center; mixed pediatric/adult
Kobbernagel et al. 2020-BESTCILIA Lancet Respir Med	Phase 3 double-blind RCT (placebo-controlled)	*n* = 90 (randomized)	7–50 yrs	Number of antibiotic courses (oral or IV) for respiratory symptoms during 6-month treatment period	Azithromycin reduced exacerbation rate by 55% vs placebo (0.44 vs 0.98 courses/6 mo; *p* = 0.006). Probability of remaining exacerbation-free at 6 months significantly higher with azithromycin. First high-quality PCD RCT demonstrating pharmacotherapy benefit	Smaller than planned sample; 6-month duration only; no data on antimicrobial resistance; action-based definition; results may not generalize to all genotypes
Singer et al. 2021 Thorax	Prospective multicenter cohort	*n* = 90	3–41 yrs (children + adults)	Any acute change in respiratory symptoms leading to treatment change (including PEx); prospectively recorded	Each 1-unit LCI increase associated with 13% greater PEx risk (HR 1.13, 95% CI 1.04–1.23). Transition from normal to abnormal LCI increased PEx risk by 87% (HR 1.87). LCI rises during PEx and may improve with treatment	LCI testing limited to specialist centers; observational; varying follow-up duration; small numbers at extremes of age
Zhou et al. 2023 Microbiol Spectr	Retrospective cross-sectional study	*n* = 72	Children + adults (median 11.3 yrs)	Increased respiratory symptoms with antibiotic initiation vs. stable (comparator)	Reduced airway microbiome diversity during PEx compared to stable state; increased *Pseudomonas aeruginosa* abundance during exacerbations. First detailed microbiome characterization of PEx state in PCD	Heterogeneous sample collection (BALF + sputum); small numbers; Chinese pediatric center; cross-sectional limitation
Gatt et al. 2023-iPEx study Pediatr Pulmonol	Retrospective cohort	52 iPEx events (28 pts)	Children (median 11.2 yrs)	Increased respiratory symptoms treated with IV antibiotics	78% (32/41) recovered to ≥ 90% baseline FEV_1_ at discharge. Most recovery occurred in first week of IV therapy. Non-response associated with lower BMI *z*-score and lower baseline FEV_1_	Retrospective; single Canadian center; pediatric-only; IV treatment defined; small event number
Ringshausen et al. 2024-CLEAN-PCD Lancet Respir Med	Phase 2 double-blind crossover RCT	*n* = 123	≥ 12 yrs	Not a primary PEx endpoint; exacerbation rate tracked as safety outcome	ENaC blocker idrevloride + HTS produced modest but statistically significant FEV_1_ improvement vs HTS alone (+ 31 mL, *p* = 0.043). No significant difference in QOL. Provides proof-of-concept for airway rehydration strategy	Short duration (28 days); underpowered for exacerbation outcomes; PEx not primary endpoint; mostly adolescent/adult; crossover limits exacerbation rate interpretation
Gatt et al. 2025-oPEx study Ann Am Thorac Soc	Retrospective cohort	297 oPEx events (85 pts)	Pediatric (mean 12.5 yrs)	Increased respiratory symptoms + physician decision to treat with oral antibiotics (oPEx)	Mean FEV_1_ fell from 85.1% to 69.5% predicted at day 1 of PEx. Recovery to baseline in 73% at 3 months, 84% at 1 year. 37% of oPEx not associated with ≥ 1 0% FEV_1_ decline. Younger age and prior non-response predicted non-recovery	Retrospective; single Canadian center; pediatric only; requires spirometry so youngest patients excluded
Gatt et al. 2025- Ann Am Thorac Soc	Retrospective cohort	*n* = 110 (pediatric)	Pediatric patients	Increased respiratory symptoms + physician decision to treat with systemic antibiotics	Late diagnosis (≥ 8 yrs) associated with significantly higher PEx rate (1.95 vs 0.75/yr, *p* < 0.01)	Single Canadian center, retrospective, pediatric only
Schreck et al. 2026- Living with PCD ERJ Open Res	International prospective cohort (longitudinal)	*n* = 660 (17,853 questionnaires)	Children, adolescents, adults	Self-reported increased respiratory symptoms in past 7 days (online questionnaire)	PEx incidence 3.1/person/year; minor variation across age groups. Higher risk in adult females (IRR 2.0) and Pseudomonas colonized patients (children IRR 1.9; adults IRR 1.4). Only 13% of exacerbation-weeks involved a health professional. *P. aeruginosa* was the most frequently isolated pathogen during PEx in adults/adolescents (44% of samples) and children (15% of samples)	Self-reported; selection bias (engaged patients); no spirometry at exacerbations; definition not validated against Lucas 2019 criteria; European-predominant cohort

*BMI* body mass index, *BALF* broncho-alveolar lavage fluid, *CT* computed tomography, *ENaC* epithelial sodium channel, *FEV*_*1*_ forced expiratory volume in 1 s, *HTS* hypertonic saline, *HR* hazard ratio, *iPEx* intravenously treated pulmonary exacerbation, *IRR* incidence rate ratio, *IV* intravenous, *LCI* lung clearance index, *oPEx* orally treated pulmonary exacerbation, *PCD* primary ciliary dyskinesia, *PEx* pulmonary exacerbation, *QOL* quality of life, *RCT* randomized controlled trial

The BESTCILIA trial (Kobbernagel 2020) was the first phase 3 RCT to demonstrate pharmacotherapy efficacy in PCD and represents the highest level of evidence for PEx prevention to date. The CLEAN-PCD trial (Ringshausen 2024) is the first PCD-specific RCT to demonstrate benefit of an airway rehydration strategy

Inflammatory marker studies and studies of CF or non-CF bronchiectasis populations only are excluded

## Incidence and frequency

Robust prospective data on PEx incidence in PCD have emerged from the international *Living with PCD* cohort study. Among 660 participants contributing nearly 18,000 follow-up questionnaires over 2 years, 1026 exacerbations were recorded, corresponding to an incidence rate of approximately 3.1 PEx per person-year with only minor variation across age groups [10]. The study further identified subgroups at higher risk, including adult females and individuals with *Pseudomonas aeruginosa* colonization, suggesting that both biological and microbiological factors influence exacerbation susceptibility.

Another international prospective study assessing 271 individuals with genetically confirmed diagnosis (248 with complete annual records) evaluated three different definitions for PEx: (1) Lucas et al. international consensus; (2) change/start of systemic antibiotics; and (3) self-reported PEx. They found that approximately 80% experienced at least one PEx per year, as assessed by the three definitions used. Furthermore, female sex and autumn season were associated with higher number of PEx, independent of the definition used. Self-reported median PEx per year was higher (2 PEx, IQR 1–5) compared to the other two definitions [24]. These findings demonstrate the importance of the definition used in capturing the exacerbation burden of PCD.

For context, in CF, approximately 35% of patients experience at least one pulmonary exacerbation annually [25] and a mean annual rate of 1–3 PEx has been reported [26, 27]. However, with the introduction of highly effective CFTR modulator therapy, recent reports show a decrease in PEx incidence [28], a trend that currently is not anticipated as no direct disease modifying PCD therapy is available.

Furthermore, late diagnosis (beyond 8 years of age) has also been identified as a risk factor, with affected patients experiencing approximately twice the annual exacerbation rate of those diagnosed at the typical age of 3–8 years [29]. PEx occurs even in the earliest years of life. Kumar et al. reported that among 35 infants diagnosed within the first 3 months of life, 66% experienced outpatient PEx within their first 2 years, with half experiencing recurrent events. By age 2, 30% had suffered a severe PEx requiring hospitalization and intravenous antibiotics [30].

These findings confirm that PEx represent a frequent and persistent burden across the lifespan in PCD and should be considered a central therapeutic target rather than an incidental complication [9, 10].

## Pathophysiology

PCD causes failure of mucociliary clearance due to dysfunctional or immotile cilia [31]. Defective ciliary fluid regulation further impairs mucus transport [32]. Mucus retention promotes bacterial colonization and an intensifying neutrophilic inflammatory response; during PEx, this inflammatory cascade accelerates, directly damaging airway structures and driving the progressive development of bronchiectasis [17]. Although CF reaches this same inflammatory endpoint through a distinct mechanism, as CFTR dysfunction causing airway surface liquid dehydration rather than a primary ciliary motility defect, the CF airway has served as a valuable model for understanding neutrophil-dominated lung injury [33].

Studies in CF have established that neutrophil elastase (NE) is detectable from early infancy, predicts bronchiectasis development, and correlates with long-term lung function decline [34, 35], providing a mechanistic framework directly applicable to PCD. This CF-derived understanding of neutrophil elastase biology has directly informed the therapeutic rationale for anti-protease and DPP1-inhibitor strategies now being trialed in bronchiectasis and PCD (see “Future directions and emerging therapies,” below); however, the comparatively more complete inflammatory resolution observed in PCD suggests that the magnitude and durability of benefit from these CF-derived approaches may differ in PCD, and PCD-specific evidence should not be assumed from CF data alone. Importantly, direct comparison of airway inflammation during PEx in both diseases shows that while neutrophilic inflammation intensifies in PCD during exacerbations, NE activity persists at higher levels in CF airways at end of treatment, suggesting more complete inflammatory resolution in PCD, with potential therapeutic implications [17, 36]. An important multicenter observational study of the airway inflammation in pediatric patients by Sagel et al. found that sputum inflammatory markers such as NE, interleukin (IL)−1β, IL-8, and tumor necrosis factor-α concentrations correlated positively with abnormal computed tomography findings and negatively with lung function. Furthermore, sputum NE, IL-1β, and tumor necrosis factor-α concentrations were higher in those with positive sputum cultures for common bacterial pathogens [37]. The increase in understanding of airway inflammation and defining specific biomarkers of airway inflammation in PCD opens the possibility for targeted therapies addressing specific components of the inflammatory response.

Moreover, this ongoing inflammatory state damages the bronchial epithelium and submucosa, impairing barrier integrity and progressively enlarging the airways, creating the hallmark bronchiectasis changes [3, 36]. The resulting structural damage further predisposes mucus retention and recurrent infection, reinforcing the self-perpetuating cycle of infection, inflammation, and airway injury.

Bacterial colonization follows a broadly age-dependent trajectory in PCD. In early childhood, sputum cultures typically yield *Haemophilus influenzae*, *Streptococcus pneumoniae*, and *Staphylococcus aureus* [38]. With advancing age and disease severity, *Pseudomonas aeruginosa* becomes increasingly prevalent. Chronic *P. aeruginosa* is associated with worse lung function, structural damage, and more frequent exacerbations [39–41]. Recent microbiome studies show reduced diversity during exacerbations with increased *Pseudomonas* abundance [42].

## Clinical impact of pulmonary exacerbations

### Impact on pulmonary function

PEx are associated with clinically significant short-term declines in lung function, and incomplete recovery may contribute to the progressive forced expiratory volume in 1 s (FEV_1_) decline observed in longitudinal PCD cohorts [43, 44]. However, a decrease in FEV_1_ is not universally observed and is therefore not included in most PEx definitions. This is supported by data from an oral-treated exacerbation (oPEx) cohort of 297 events, in which 37% of exacerbations were not associated with a ≥ 10% decline in FEV_1_ [45]. Comparable findings have been reported in CF, where up to 50% of exacerbations do not result in significant spirometric decline [46], reinforcing the notion that FEV_1_ reduction should not be a mandatory diagnostic criterion.

Three studies have now characterized lung function trajectories following PEx in PCD [8, 18, 45]. All defined PEx as changes in baseline respiratory symptoms requiring systemic antibiotic treatment. For severe exacerbations treated with intravenous antibiotics (iPEx), a pediatric cohort of 52 iPEx demonstrated that most lung function recovery occurs within the first week of therapy, with 78% of patients recovering to at least 90% of baseline FEV_1_ by discharge [8]. Lower body mass index* z*-score and lower pre-exacerbation FEV_1_ were associated with poorer recovery. Similar findings were reported by Sunther et al., with 23/30 (77%) of children with iPEx recovered to baseline lung function within 3 months [18].

While IV-treated exacerbations have been the primary focus, the natural history of milder, orally treated pulmonary exacerbations (oPEx) has recently been characterized in a larger dataset of 297 oPEx events across 85 pediatric PCD patients [45]. Mean FEV_1_ fell from 85.1 to 69.5% predicted during PEx (absolute decline of 10.4%, relative decline of 12.9%), indicating that oral-treatment events cause substantial acute functional loss. Recovery rates were 73% at 3 months post-exacerbation and increased to 84% at 1 year, implying that approximately one in six PEx treated in the community resulted in permanent, unrecovered lung function loss. Risk factors for non-recovery included younger age and prior non-response, suggesting the presence of a subgroup of patients with a consistently impaired recovery phenotype. When considering complete recovery (≥ 100% of baseline), response rates were even lower, consistent with findings in CF [46].

The long-term impact of PEx on lung function decline in PCD remains uncertain. However, the negative impact may be inferred from CF data, as in CF approximately 50% of the long-term FEV_1_ decline has been attributed to severe PEx events [47]. These data carry direct clinical implications: not only should PEx be treated promptly and effectively, but post-exacerbation spirometry reassessment should be standard practice to identify non-responders who may benefit from extended treatment or altered management strategies, to prevent ongoing decline in lung function.

### Lung clearance index as a predictor and outcome measure

The lung clearance index (LCI), derived from multiple-breath washout testing, has emerged as a sensitive indicator of ventilation inhomogeneity in early and mild lung disease in PCD, detectable when spirometry is still normal, and may therefore provide complementary information during disease monitoring [48]. A multicenter cohort study of 90 PCD patients demonstrated that each one-unit increase in LCI was associated with a 13% increase in the risk of future PEx (hazard ratio 1.13, 95% CI 1.04–1.23), and that transition from a normal to an abnormal LCI increased future exacerbation risk by 87% (HR 1.87, 95% CI 1.08–3.23) [49]. During PEx, LCI increases significantly and may improve with treatment, suggesting that it may serve as both a predictive biomarker and a responsive outcome measure in clinical trials. However, its long-term utility in tracking PCD lung disease remains unclear [50]. Furthermore, its limited availability outside specialized centers currently restricts widespread clinical use, although efforts are underway to address this limitation with international consensus recommendations now providing standardized protocols for equipment specifications, test performance, and analysis [51] and improving accessibility for LCI testing at the community setting [52].

### Disease severity and structural consequences

PEx frequency is strongly associated with disease severity. Patients with two or more exacerbations per year compared with fewer show significantly lower FEV_1_, more lobes involved on computerized tomography (CT), worse bronchiectasis severity index scores including modified Bhalla CT score, and a higher prevalence of chronic *P. aeruginosa* colonization (33.3% versus 13.6%) [39]. Multivariable analyses confirm that PEx frequency and *P. aeruginosa* colonization are independently associated with structural and functional severity markers, not merely confounders of each other but are independently linked to various respiratory severity outcomes. These relationships argue for integrated management of both exacerbation burden and pathogen acquisition as dual targets in PCD care.

## Management of acute pulmonary exacerbations

**Fig. 1:**
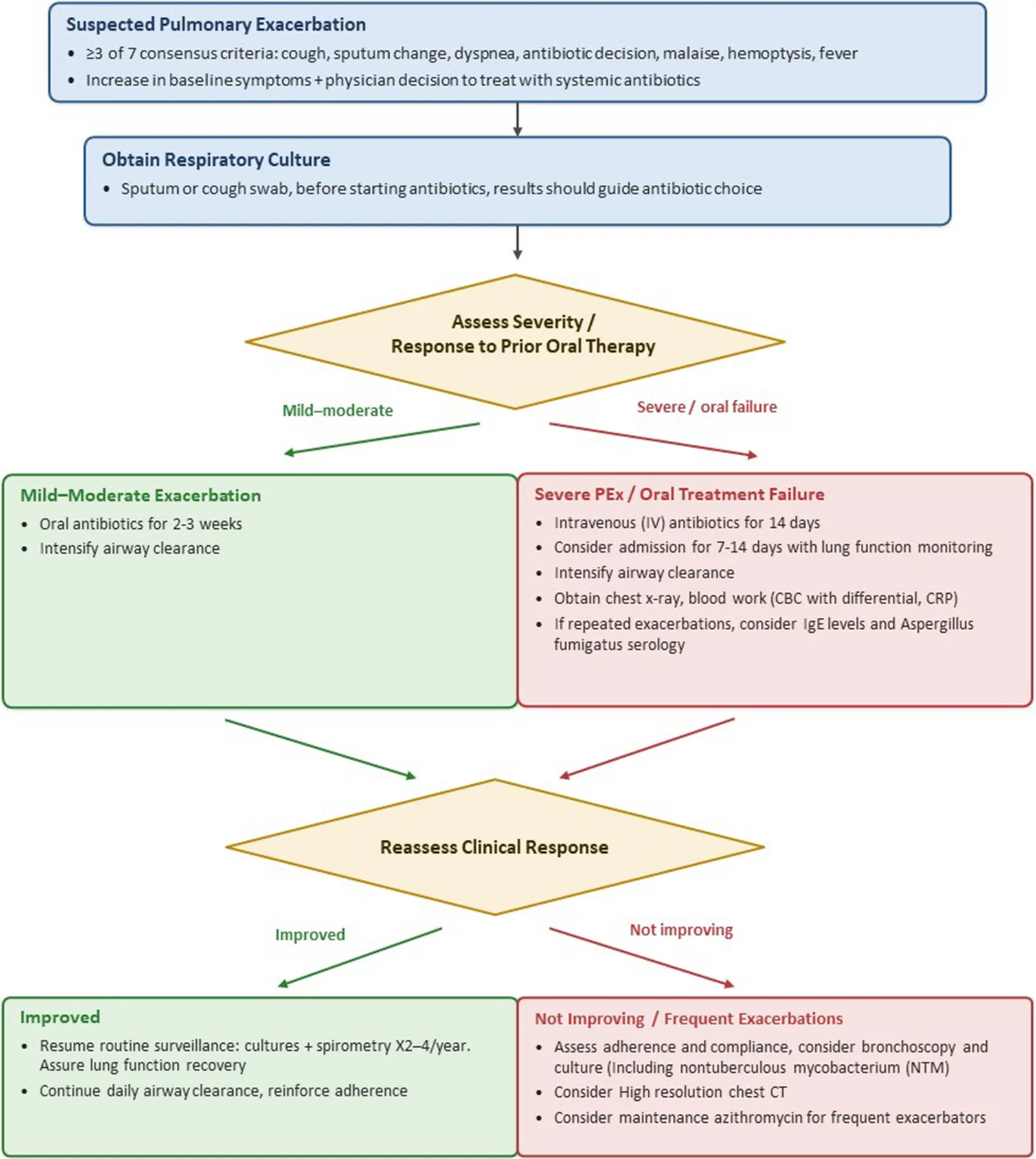
Management for pulmonary exacerbation in PCD

Management is extrapolated from CF and bronchiectasis due to limited PCD-specific trials [7, 11, 53]. The general approach includes antibiotic therapy, intensification of airway clearance, and supportive care, with treatment intensity tailored to PEx severity as outlined in the stepwise management algorithm summarized in Fig. 1.

### Antibiotic therapy

Antibiotic selection should ideally be guided by the most recent sputum microbiology, and lower airway cultures should be obtained whenever possible during PEx to optimize antimicrobial targeting. Common pathogens during PEx include *H. influenzae* and *S. aureus* [45]. Therefore, empiric oral therapy for mild-to-moderate PEx should target the predominant age-appropriate pathogens: broad-spectrum agents such as amoxicillin-clavulanate or equivalent cephalosporins are appropriate first-line choices in patients without *P. aeruginosa* [11]. Treatment duration of at least 14 days is generally recommended, with many expert guidelines suggesting courses of 2 to 3 weeks, although high-quality evidence defining optimal duration in PCD remains lacking [11, 54]. A recent international consensus statement has suggested that in PEx not responding to treatment with oral antibiotics, blood testing is recommended for C-reactive protein (CRP), differential white blood cell count and hemoglobin levels. In practice, some clinicians may use inflammatory biomarkers such as CRP to help gauge treatment response during PEx, an approach extrapolated from CF data showing that circulating CRP and other inflammatory proteins fall significantly with successful antibiotic treatment [55]. However, this strategy has not been formally validated in PCD, and its routine use for guiding treatment duration in this population remains of uncertain benefit. Additionally, a study describing severe exacerbation with intravenous treatment reported that on day 1 of admission, the median WBC, neutrophils count, and erythrocyte sedimentation rate (no CRP stated) were all in the normal range [8], further questioning the clinical utility of blood work in PEx management.

Intravenous antibiotics are indicated for PEx unresponsive to oral therapy, for those causing significant functional deterioration, and for those of clinical severity requiring hospitalization, consistent with the severe/oral failure pathway in Fig. 1 [45, 53, 54]. Data from iPEx study suggest that most lung function recovery during intravenous treatment occurs within the first week, with continued improvement during the second week, and that failure to achieve spirometric recovery by discharge identifies a higher-risk subgroup requiring closer follow-up [8].

Inhaled antibiotics, which are well established for long-term suppression of *P. aeruginosa* in CF and non-CF bronchiectasis, have limited direct evidence in PCD but are commonly applied by analogy in patients with chronic infection [53, 54]. Eradication of newly acquired *P. aeruginosa*, following non-CF bronchiectasis protocols, is recommended by expert consensus [11], and has shown promising results in retrospective PCD studies [56, 57].

### Airway clearance and mucoactive agents

Intensification of airway clearance therapy (ACT) is a cornerstone of PEx management and should be commenced at symptom onset and maintained beyond the antibiotic course [45, 58]. Techniques include oscillatory positive expiratory pressure devices, active cycle of breathing, autogenic drainage, and postural drainage [59, 60]. Importantly, cough clearance is generally preserved in PCD and supports the effectiveness of airway clearance strategies [11]. Inhaled hypertonic saline may enhance mucus hydration and facilitate clearance through physiological rationale. The first PCD-specific randomized trial of inhaled hypertonic saline (*n* = 24 adults, 12 weeks) showed no improvement in FEV_1_ or overall quality of life, with only one secondary outcome, the QoL-B Health Perception scale, reaching significance, and a higher frequency of mild adverse events [61]. Subsequently, the CLEAN-PCD trial (detailed below) demonstrated a modest but statistically significant FEV_1_ benefit when combining an ENaC blocker with hypertonic saline versus hypertonic saline alone, without significant quality-of-life improvement [62]. Taken together, robust evidence supporting long-term clinical benefit of inhaled hypertonic saline in PCD remains lacking, though it is widely used in clinical practice [63].

The role of recombinant human DNase (rhDNase) remains uncertain. In non-CF bronchiectasis, rhDNase has been associated with worse outcomes, including increased PEx frequency and greater FEV_1_ decline [64]. However, given the distinct pathophysiology of PCD, direct extrapolation may not be appropriate. A small observational study in adults with PCD reported a reduction in PEx frequency following 6 months of rhDNase, without significant change in lung function [65]. Despite this limited and inconclusive evidence, a recent North American survey found that approximately 30% of patients at specialized PCD centers are treated with rhDNase [63], highlighting a significant gap between clinical practice and the evidence base. PCD-specific randomized trials evaluating mucoactive therapies are needed to guide these decisions, specifically in patients with severe disease and with higher burden of baseline symptoms.

### Hospitalization, infection control, and systemic steroids

Decisions regarding hospitalization should balance clinical severity with the risk of nosocomial cross-infection. The BEAT-PCD consensus recommends that patients be segregated in clinical settings, particularly those colonized with *P. aeruginosa* or resistant organisms [66]. Where clinically feasible, home intravenous antibiotic therapy may provide an effective alternative, maintaining treatment intensity while reducing hospital exposure [66].

The role of systemic corticosteroids during PEx remains unclear. Although PEx are characterized by heightened airway inflammation, and bronchodilator reversibility is common in PCD independent of asthma diagnosis or the presence of atopy [67, 68], systemic corticosteroids are not routinely recommended during PEx. This lack of recommendation reflects a combination of factors: the complete absence of PCD-specific efficacy data, CF trial evidence failing to demonstrate consistent benefit on lung function recovery [69, 70], and the well-established adverse-effect profile of systemic corticosteroids that are particularly relevant given the recurrent, lifelong nature of PEx in PCD. PCD-specific trials are needed before any recommendation can be made.

## Evidence from randomized controlled trials

Until recently, the management of PCD was based almost entirely on extrapolated evidence. However, several randomized controlled trials have now begun to define a PCD-specific evidence base for PEx outcomes.

### BESTCILIA trial (2020)

The BESTCILIA (Better Experimental Screening and Treatment for Primary Ciliary Dyskinesia) trial was the first multicenter, double-blind, randomized, placebo-controlled phase 3 trial to demonstrate efficacy of a pharmacotherapy in PCD [22]. Conducted across six European centers, it randomized 90 patients aged 7–50 years with confirmed PCD to azithromycin or placebo for 6 months. The azithromycin group experienced a significant 55% reduction in exacerbation frequency. Additionally, the probability of remaining exacerbation-free at 6 months was significantly higher in the treatment group. Treatment was generally well tolerated, although gastrointestinal side effects were more frequent with azithromycin. This trial represents a landmark advance in PCD management, providing the first high-quality evidence supporting maintenance macrolide therapy in patients with frequent PEx. However, limitations include a smaller-than-planned sample size and lack of long-term data on antimicrobial resistance.

### CLEAN-PCD trial (2024)

The CLEAN-PCD trial evaluated the epithelial sodium channel (ENaC) blocker idrevloride, designed to improve airway hydration, in combination with hypertonic saline [62]. Mechanistically, this block will reduce sodium and fluid hyperabsorption across the airway epithelium, and is intended to restore airway surface liquid volume and improve mucus hydration and clearance, addressing a pathophysiological mechanism relevant to PCD independent of the primary ciliary defect. This phase 2, randomized, double-blind crossover trial included 123 patients aged ≥ 12 years. The combination therapy resulted in a modest but statistically significant improvement in FEV_1_ compared with hypertonic saline alone. Improvements were consistent across lung function measures, although quality-of-life outcomes did not show significant change.

The relatively short treatment duration (28 days) limited assessment of PEx outcomes, and the magnitude of lung function improvement was modest. Nevertheless, the trial provides important proof-of-concept evidence supporting airway rehydration as a therapeutic strategy in PCD and justifies longer-term studies with PEx endpoints.

### REPEAT trial

The REPEAT (Reducing Exacerbations in Primary Ciliary Dyskinesia with Azithromycin and/or Erdosteine Treatment; ACTRN12619000564156) trial was designed as an Australian multicenter trial with a 12-month intervention period, placebo-controlled RCT enrolling patients with at least two PEx in the preceding 18 months [23]. At the time of writing, results have not yet been published. Given the established benefit of Erdosteine in non-CF bronchiectasis, this trial has the potential to further expand the therapeutic options available for PCD and to determine whether combination therapy offers additive benefit.

## Prevention strategies

Prevention of PEx is a central goal in the long-term management of PCD. Daily airway clearance physiotherapy is universally recommended and should be performed at least twice daily, with increased intensity during PEx [11, 54]. Airway clearance techniques (ACT) include modified postural drainage, percussion and expiratory vibration, positive expiratory pressure (PEP) with or without oscillating (OPEP), active cycle of breathing, autogenic drainage, high-frequency chest wall oscillation vest, and physical activity/aerobic exercise. Bingol et al. completed a randomized crossover trial comparing OPEP vs conventional chest physiotherapy (CPP). This study enrolled 30 patients aged 6–20 years, with two 3-month treatment periods separated by a 15-day washout and found no significant difference between OPEP and CPP in pulmonary function measures or PEx rate and time period until the first PEx, although both methods showed improvement in lung function compared to baseline [71]. However, comfort of use was significantly higher in the OPEP group compared to CPP. Nonetheless, the decision on type of ACT should be based on patient preference, age, local availability, insurance coverage, and by the physiotherapy team expertise [59].

Regular aerobic exercise is also encouraged, as it enhances mucociliary clearance and supports overall respiratory health [72]. Vaccination against influenza, pneumococcus, pertussis, and *Haemophilus influenzae* type b is recommended, along with COVID-19 vaccination given the increased risk of severe lower respiratory tract disease in patients with structural lung disease [11, 73, 74].

Management of upper airway disease is an important component of PEx prevention. Functional endoscopic sinus surgery has been shown to reduce PEx frequency and improve lung function in selected patients, reflecting the “united airways” concept linking upper and lower respiratory tract disease [75]. Environmental exposures should be minimized. Cigarette smoke and indoor air pollution impair mucociliary clearance and exacerbate airway inflammation [11]. Increasing use of electronic cigarettes is particularly concerning; these devices have been shown to reduce ciliary beat frequency and impair epithelial function [76, 77]. Notably, a small study in adolescents with PCD reported e-cigarette use in 19% of participants, highlighting a clinically relevant and underrecognized risk behavior [78].

Maintenance macrolide therapy should be considered in patients with three or more PEx per year, supported by the BESTCILIA trial evidence [22]. Monitoring macrolide resistance in commensal and pathogenic bacteria during long-term therapy is important, including nontuberculous mycobacteria cultures, particularly in view of the theoretical risk of selecting resistant organisms with prolonged use.

## Future directions and emerging therapies

The therapeutic pipeline for PCD is expanding beyond antibiotics and physiotherapy, with several mechanistic approaches under active investigation [79, 80].

Anti-inflammatory strategies targeting neutrophilic airway inflammation, the dominant effector mechanism of progressive lung damage in PCD, include dipeptidyl peptidase 1 (DPP1) inhibitors and neutrophil elastase inhibitors, both of which have demonstrated clinical benefit in reducing PEx in non-CF bronchiectasis and may have similar benefits in PCD [80–82]. Those various emerging therapies are primarily focusing on reducing the chronic bacterial burden that drives neutrophilic inflammation and attenuating the same neutrophilic inflammatory cascade, offering a multifaceted approach to the excessive neutrophilic inflammation that characterizes PCD exacerbations.

Novel anti-infective approaches include inhaled antibiotics for long-term bacterial suppression, *P. aeruginosa*–directed antibodies and vaccines, and inhaled immunoglobulin G [80]. Mucolytic strategies, beyond hypertonic saline [61], are under investigation.

Perhaps the most transformative area is genetic therapy. Given the genetic heterogeneity of PCD, with more than 60 implicated genes and over 1200 variants [83], multiple approaches will likely be required [79, 80].

The development of validated outcome measures is also critical. The lung clearance index shows promise as a sensitive biomarker [49], although its availability is currently limited. Exhaled breath analysis using volatile organic compounds (VOCs), metabolic byproducts released by airway microorganisms, represents another emerging noninvasive approach [84]. Mass spectrometry–based studies have identified a range of VOCs with potential utility for detecting respiratory infections and monitoring PEx in PCD [85], though clinical validation is still in early stages.

Patient-reported outcomes, including QOL-PCD instruments, will be essential for capturing the patient perspective and for regulatory approval of new therapies [86, 87]. International registries provide essential infrastructure for large-scale natural history studies and platform trial designs that can overcome the recruitment challenges inherent to this rare disease.

## Conclusions

Pulmonary exacerbations are a central and clinically significant feature of PCD across all age groups, contributing to progressive lung disease, structural airway damage, and impaired quality of life. Recent advances have substantially improved our understanding of exacerbation epidemiology, lung function trajectories, and treatment response, and landmark trials such as BESTCILIA and CLEAN-PCD have begun to establish PCD-specific evidence base where none previously existed.

Important gaps nonetheless remain. A universally accepted and prospectively validated definition of PEx is still lacking, and uncertainties persist regarding optimal antibiotic strategies, predictors of incomplete lung function recovery, and the role of anti-inflammatory and targeted therapies in both acute management and prevention.

For the practicing clinician, key principles include early recognition and prompt treatment of exacerbations, microbiology-guided antibiotic selection, intensification of airway clearance, and post-exacerbation spirometry reassessment to identify non-responders. Maintenance therapies should be considered in high-risk patients, and vigilance for *P. aeruginosa* acquisition remains essential. Continued international collaboration and investment in PCD-specific research will be critical to translating these emerging insights into improved long-term outcomes for patients with this rare but impactful disease.

## Data Availability

No datasets were generated or analysed during the current study.
